# Determination of the binding affinities of *Neisseria meningitidis* serogroup W capsule polymerase with two nucleotide sugar substrates

**DOI:** 10.1186/s13104-018-3596-y

**Published:** 2018-07-16

**Authors:** Abeer Sharyan, Cendy Gonzalez, Ophelia Ukaegbu, Kayla Powell, Pumtiwitt C. McCarthy

**Affiliations:** 1 Department of Chemistry, Morgan State University, 1700 East Cold Spring Lane, Baltimore, MD 21251 USA; 2 Department of Biology, Morgan State University, 1700 East Cold Spring Lane, Baltimore, MD 21251 USA

**Keywords:** *Neisseria meningitidis*, Glycosyltransferases, Enzyme kinetics, Vaccine development

## Abstract

**Objective:**

Meningococcal meningitis is a public health burden. Immunization strategies have reduced global incidence of the disease. Glycoconjugate vaccines are the most effective type of vaccine to combat most causes of meningococcal meningitis. These vaccines contain capsular polysaccharide fragments from disease-causing serogroups of *Neisseria meningitidis* that are chemically attached to a carrier protein. The enzymes responsible for capsular polysaccharide synthesis can serve as tools to make these critical vaccine components. One such enzyme is the *N. meningitidis* serogroup W capsule polymerase. This enzyme is responsible for creating the galactose-sialic acid containing capsular polysaccharide of this serogroup. Our aim in this study was to determine the binding affinities of nucleotide sugar donors CMP-sialic acid and UDP-galactose using a coupled transferase assay to inform future work to modulate polysaccharide synthesis by this enzyme.

**Results:**

We determined a K_m_ of 66.8 µM for CMP-sialic acid and a K_m_ for UDP-galactose of 3.9 µM. These values are lower than reported values for other retaining galactosyltransferases and inverting sialyltransferases respectively. There were difficulties obtaining reliable data for galactosyltransferase activity. An alternate strategy is needed to assess kinetic parameters of the separate transferase activities for this enzyme.

**Electronic supplementary material:**

The online version of this article (10.1186/s13104-018-3596-y) contains supplementary material, which is available to authorized users.

## Introduction

*Neisseria meningitidis* is a leading cause of bacterial meningitis. Vaccines have helped to curb the spread of disease [1, 2]. Capsular polysaccharides surrounding the bacteria are a virulence factor [3]. Glycoconjugate vaccines are one of the most effective vaccine types and contain capsular polysaccharide fragments attached to a carrier protein [4]. While polysaccharide fragments isolated from the producing organism can be used for vaccine production, these are typically heterogenous. Serogroup-specific glycosyltransferase enzymes from *Neisseria* can serve as an alternative and potentially controllable method to obtain capsular polysaccharides for production of homogeneous vaccines [5–14]. Knowledge of the kinetic parameters of these glycosyltransferases will facilitate their use in this way. There are a few papers that describe these parameters for some enzymes but not all of these [5, 7, 14, 15]. This work focuses on kinetic analysis of one of these polysaccharide-producing enzymes, the *N. meningitidis* serogroup W capsule polymerase, to address this gap in knowledge.

The *N. meningitidis* serogroup W capsule polymerase is a 120 kDa protein that synthesizes the galactose-sialic acid polymer that surrounds *N. meningitidis* serogroup W [9, 12]. Each polymer contains a unit in which the carbon 1 of galactose is linked through an α-glycosidic linkage to carbon 4 of sialic acid. This unit is repeated via an α-glycosidic linkage between carbon 2 of sialic acid and carbon 6 of galactose. The enzyme contains three domains: an amino-terminal galactosyltransferase domain, an intervening sequence, and a carboxyl-terminal sialyltransferase domain. This paper describes our efforts to determine previously unknown kinetic parameters of nucleotide donor sugars (CMP-sialic acid and UDP-galactose) with this enzyme using a multi-enzyme coupled activity assay [16]. These results expand the limited characterization of this enzyme [9, 12, 17]. One long-term goal is to determine key amino acids for catalysis and substrate binding, which can be exploited to modulate polysaccharide synthesis. Thorough understanding of the kinetic parameters of the *N. meningitidis* serogroup W capsule polymerase will provide important fundamental knowledge to guide future use of this enzyme as a chemoenzymatic tool for vaccine production.

## Main text

### Methods and materials

All reagents obtained from Sigma-Aldrich unless stated otherwise.

#### Expression and growth of the *N. meningitidis* serogroup W capsule polymerase

A plasmid containing the capsule polymerase, pStrep-W135-His (a generous gift of Dr. Willie Vann, FDA/CBER) was transformed into *E. coli* KRX cells (Promega) and plated on plates containing ampicillin (100 µg/mL) overnight. LB-Amp media (3 mL) was used to resuspend cells and this resuspension was used to inoculate a 1 L culture of LB-Amp media. Growth was induced with 20% rhamnose and IPTG (0.5 mM final) according to manufacturer’s instructions. Cells were grown overnight and harvested by centrifugation (Beckman-Coulter J25I) at 6500 rpm for 15 min.

#### Protein purification of the *N. meningitidis* serogroup W capsule polymerase

Purification was performed similar to a published procedure with specific modifications outlined below [9, 12]. Bacterial cells were sonicated in 50 mM Tris, 300 mM NaCl pH 8.0 in the presence of protease inhibitor (Roche). Sonication was performed on ice 10× for 10 s with 1 min rest. Lysozyme (3 mL of a 10 mg/mL lysozyme stock solution in 50 mM Tris, 50 mM MgCl_2_, pH 8.0) was added to the solution and placed on ice for 1 h. After sonication, the lysate was centrifuged at 10,000 rpm for 15 min. The supernatant was incubated with Ni^2+^ resin (Qiagen) at 4 °C. The resin was returned to a column and washed with 5 CV of 50 mM Tris, 300 mM NaCl, 50 mM imidazole pH 8.0. Protein was eluted using 50 mM Tris, 300 mM NaCl, 150 imidazole, pH 8.0. Collected fractions were tested by Bradford reagent and purity assessed on a 4–12% Bis–Tris SDS-PAGE gel (Life Technologies) (Additional file 1: Figure S1).

#### Acid hydrolysis of *N. meningitidis* serogroup W polysaccharide

The polysaccharide was hydrolyzed similar to a published procedure [14, 15]. A solution (500 µL of 10 mg/mL) of *N. meningitidis* serogroup W polysaccharide (a generous gift of Dr. Willie Vann, FDA/CBER) dissolved in water was diluted with 500 µL of 1% acetic acid in a 10 × 100 mm polypropylene tube. The tube was covered and heated at 80 °C for 1 h on a heat block. The tube was then returned to room temperature for 5 min and then added to ice for 10 min. The solution was transferred to a microcentrifuge tube and dried by SpeedVac. The sample was reconstituted in 500 µL of distilled water.

#### Absorbance-based multi-enzyme coupled activity assay

The absorbance-based assay was performed according to a previously used method [5, 14, 15]. The effect of varying nucleotide donor concentrations on activity was investigated. The methods described here differ mainly from those described in the Additional file 2: Methods in the number of replicates (3 vs. 2), the pyruvate kinase (PK) and lactate dehydrogenase (LDH) used (2 enzyme solutions vs. a combined enzyme solution) and the time of equilibration at 25 °C before readings began (10 min vs. 5 min). Reactions were performed with serogroup W oligosaccharide acceptor (625 µg/mL), 0.7 mM PEP, 2 mM ATP, 2.1 units of PK, 10.3 units of LDH, 0.2 mM NADH, CMP-sialic acid (Nacalai Tesque) (at 10, 20, 40, 80, 160, 320, 640 µM or steady at 1 mM), UDP-galactose (at 10, 20, 40, 80, 160, 320, 640 µM or steady at 1 mM), 1 mM DTT, with or without 0.05 units/mL NMPK in 50 mM Tris, 25 mM MgCl_2_ (pH 8.0). All reactions were equilibrated for 10 min and transferred to a cuvette for absorbance readings at 340 nm using a Cary 50 spectrophotometer. The background rate was monitored for 5 min after which the serogroup W capsule polymerase (25 µg/mL final concentration) was added. Absorbance was monitored for 10 additional min. All reactions were performed at room temperature in triplicate in a final volume of 200 µL. The concentration of the oxidized NAD produced was calculated using the following equation (((Slope − Background)/2)/6220 M^−1^ cm^−1^) × 10^6^. The rates of each reaction were calculated and plotted using GraphPad Prism.

### Results and discussion

#### The multi-enzyme coupled transferase assay for measuring capsule polymerase activity

**Fig. 1:**
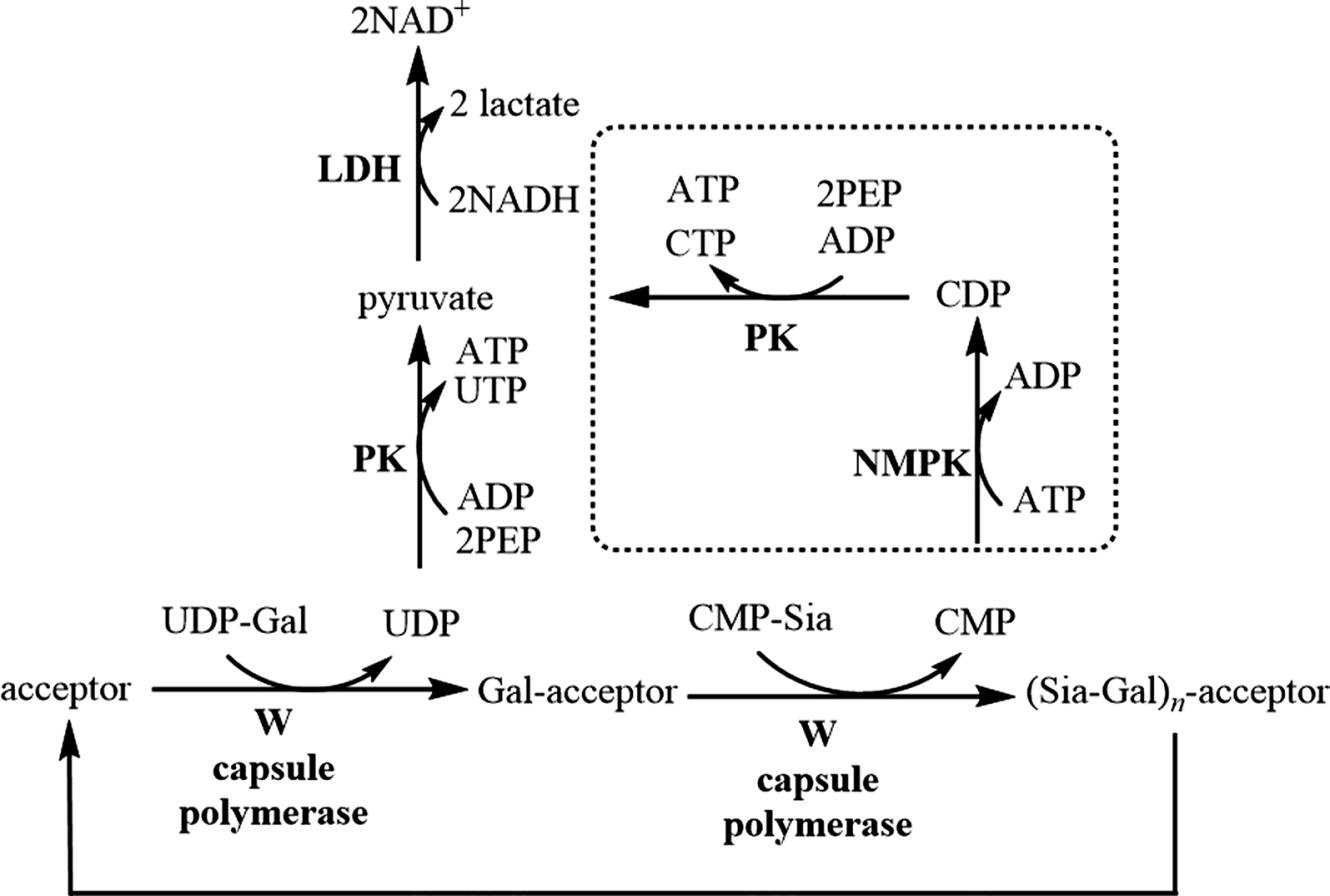
Schematic of the multi-enzyme coupled transferase assay. UDP-galactosyltransferase activity of the serogroup W capsule polymerase can be measured separately from sialyltransferase activity. The boxed reactions indicate reactions that take place in the presence of NMPK

To measure enzymatic activity of the *N. meningitidis* serogroup W capsule polymerase, we used an assay that links glycosyltransferase activity to NADH oxidation, which can be monitored by measuring the decrease in absorbance at 340 nm (Fig. 1). This assay has been used successfully to monitor kinetics of other *N. meningitidis* capsule-producing enzymes that use a single nucleotide sugar donor [5, 14, 15]. Glycosyltransferase activity is linked to NADH oxidation through three linking enzymes: nucleotide monophosphate kinase (NMPK), pyruvate kinase (PK) and lactate dehydrogenase (LDH). The serogroup W capsule polymerase uses two nucleotide donor sugars, adding an additional layer of complexity to these studies. To study this bifunctional polymerase, a key assay component is NMPK. NMPK adds a phosphate to cytidine monophosphate (CMP) produced from the sialyltransferase reaction (Fig. 1). The resulting cytidine diphosphate (CDP) then acts as a phosphate acceptor in the next reaction with PK leading to production of pyruvate. Additionally, PK catalyzes addition of phosphate to uridine diphosphate (UDP) produced in the galactosyltransferase reaction. Finally, pyruvate resulting from both transferase reactions is reduced to lactate with concomitant production of NAD^+^ by LDH. Because of the nature of the serogroup W capsule polymerase, we envisioned monitoring the UDP-galactose activity alone by omission of NMPK from the reaction mixture. Using this method, we were able to determine the effect of varying UDP-galactose concentrations on catalytic rates however there was variability in the data.

#### Accurate determination of binding affinity of UDP-galactose hampered by data variability

Our initial studies with *N. meningitidis* serogroup W capsule polymerase used a low range of UDP-galactose concentrations (10–640 µM) on par with a previous study performed with the *N. meningitidis* serogroup X galactophosphotransferase [13, 15]. We determined a low K_m_ value (13.9 µM) however the fit of the data on the Lineweaver–Burke plot was poor (r^2^ = 0.4248) leading us to question these results (Additional file 3: Figure S2). Once repeated, the data suggested we needed to widen our range of concentrations to assure we were in a concentration range of at least five times K_m_ (results not shown). In our next iteration of these experiments we used a wider range of concentrations (20, 40, 80, 320, 640, 1280, and 2560 μM, Additional file 4: Figure S3). The K_m_ value was calculated to be 50.7 µM with removal of a potential outlier point. If this point is included, the K_m_ value is adjusted to 31.7 µM.

**Fig. 2:**
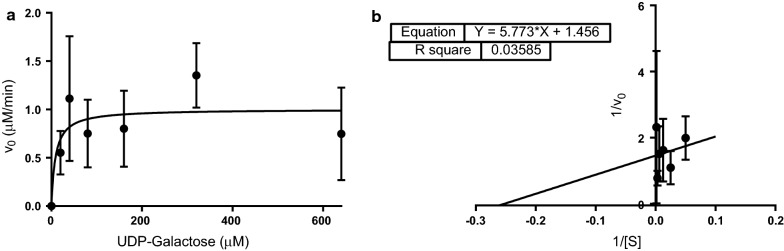
Kinetic effects of varying UDP-galactose concentrations on UDP-galactosyltransferase activity of the *N. meningitidis* serogroup W capsule polymerase. Reactions were performed in triplicate. The mean is plotted, and error bars represent standard deviation. **a** Michaelis–Menten plot and **b** Lineweaver–Burke plot

In the final iteration of these experiments, the number of replicates were increased to 3 and the concentration range was shortened to effectively determine the K_m_ value. Additionally, the equilibration time was increased to 10 min to ensure that any contaminating nucleotide donors present in the kinase solutions or any free CMP or UDP is depleted fully. However, these data (Fig. 2) show great variability. The absorbance changes observed in the absence of NMPK are much lower than those seen in the presence of NMPK which most likely impacted the data. From the data obtained, a K_m_ value of 3.9 µM was determined however the fit to this data is extremely poor.

There is no other *N. meningitidis* capsule producing enzyme that creates a galactose-containing polysaccharide to compare this value to and the kinetic parameters of the closest related enzyme (the serogroup Y capsule polymerase) has yet to be determined. In terms of polymer produced, comparisons could be made to the *N. meningitidis* serogroup X which uses a similar nucleotide donor sugar, UDP-*N*-acetylglucosamine (UDP-GlcNAc). This enzyme produces a homopolymer of (α1 → 4)-linked *N*-acetylglucosamine (GlcNAc)-1-phosphate. The K_m_ value for UDP-GlcNAc has been determined to be 46.5 µM [14]. A more apt comparison would be with a member of the same CAZY class of glycosyltransferase. The N-terminal domain of the serogroup W enzyme is categorized as GT4 which includes retaining α-glucosyl- and α-galactosyltransferases. The best characterized is the LgtC protein from *N. meningitidis* [18–20]. This protein is involved in lipooligosaccharide biosynthesis and uses UDP-galactose as a nucleotide donor sugar. A K_m_ value of 29 µM has been reported [18]. Thus, the UDP-galactose binding affinity that we measure for *N. meningitidis* serogroup W capsule polymerase is much lower than these literature examples.

#### The binding affinity for CMP-sialic acid is tighter than other *N. meningitidis* sialyltransferases

**Fig. 3:**
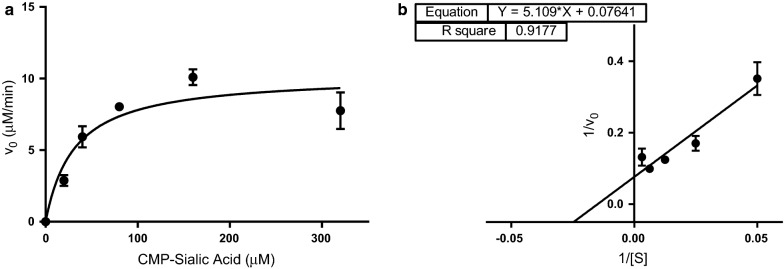
Kinetic effects of varying CMP-sialic acid concentrations on activity of the *N. meningitidis* serogroup W capsule polymerase. Reactions were performed in triplicate. The mean is plotted, and error bars represent standard deviation. **a** Michaelis–Menten plot and **b** Lineweaver–Burke plot

We also wished to investigate the binding affinity of *N. meningitidis* serogroup W capsule polymerase for its second donor substrate, CMP-sialic acid. Other disease-causing serogroups of *N. meningitidis* create capsular polysaccharides containing sialic acid with reported K_m_ values for CMP-sialic acid of 420 µM (serogroup C polysialyltransferase) and 432 µM (serogroup B polysialyltransferase) [5, 7]. However, enzymes from groups B and C have no sequence identity to group W or Y enzymes and are members of a different enzyme family so the kinetics may differ [21]. To determine binding affinity for CMP-sialic acid, we needed to include NMPK in the reaction mixture (Fig. 1). In our first iterations of these experiments, there were difficulties. The background rates for 320–2560 µM were the same or slightly higher than the reaction rates which did not allow us to determine initial rates for these concentrations. We performed these experiments again focusing on the range of 0–320 µM and increasing the equilibration time before absorbance measurements to 10 min. The data obtained showed significantly less variability than UDP-galactose allowing stronger confidence in this data (Fig. 3). The K_m_ determined for CMP-sialic acid was 66.8 µM.

The work here highlights the fact that this assay is better suited for homopolymeric glycosyltransferases. We can measure UDP-galactose binding affinity independently, however, with the inclusion of NMPK we are measuring a combined rate for both transferase activities of the serogroup W capsule polymerase. In addition, extreme variability was observed with UDP-galactose studies. We are now focused on optimizing a commercially available set of assays that will unequivocally allow us to measure independent binding affinities for both nucleotide donor sugars [22].

## Limitations

The major limitation is that while a K_m_ value for UDP-galactose was determined there is low confidence in this determination due to extreme data variability. Therefore, a full picture of transferase activity remains unclear.

## Additional files

**Additional file 1: Figure S1.** Representative SDS-PAGE gel electrophoresis of samples from purification of recombinant *N. meningitidis* serogroup W capsule polymerase. Lanes 2 and 3 are samples from the eluate. Lane 4 is the molecular weight marker containing proteins of the specified molecular weights. Lanes 5–7 are samples from the column wash steps. Lane 8 contains column flow through, Lane 9 is the cell lysate and Lane 10 is the supernatant. Lanes 1, 11, and 12 were not loaded with sample.

**Additional file 2: Methods.** Absorbance-based multi-enzyme coupled activity assay. Describes the conditions of the assay for data produced in Additional file 3: Figure S2 and Additional file 4: Figure S3

**Additional file 3: Figure S2.** Kinetic effects of varying UDP-galactose concentrations (10–640 µM) on UDP-galactosyltransferase activity of the *N. meningitidis* serogroup W capsule polymerase. Reactions were performed in duplicate and the averages are plotted. A) Michaelis-Menten plot and B) Lineweaver-Burke plot.

**Additional file 4: Figure S3.** Kinetic effects of varying UDP-galactose concentrations (20–2560 µM) on UDP– galactosyltransferase activity. This data includes the potential outlier (640 µM). Reactions were performed in duplicate and the averages are plotted. A) Michaelis-Menten plot and B) Lineweaver-Burke plot.

## Abbreviations

ATP: adenosine triphosphate
CDP: cytidine diphosphate
CMP: cytidine monophosphate
DTT: dithiothreitol
K_m_: Michaelis constant
LDH: lactose dehydrogenase
NAD^+^: oxidized form of nicotinamide adenine dinucleotide
NADH: reduced form of nicotinamide adenine dinucleotide
PEP: phosphoenolpyruvate
PK: pyruvate kinase
UDP: uridine diphosphate
UMP: uridine monophosphate
