# Supplementary figures and images for: Determination of the binding affinities of Neisseria meningitidis serogroup W capsule polymerase with two nucleotide sugar substrates
Source: BMC Res Notes. 2018 Jul 16;11:482. doi: 10.1186/s13104-018-3596-y (PMC6048754; doi:10.1186/s13104-018-3596-y)

## Slide 1
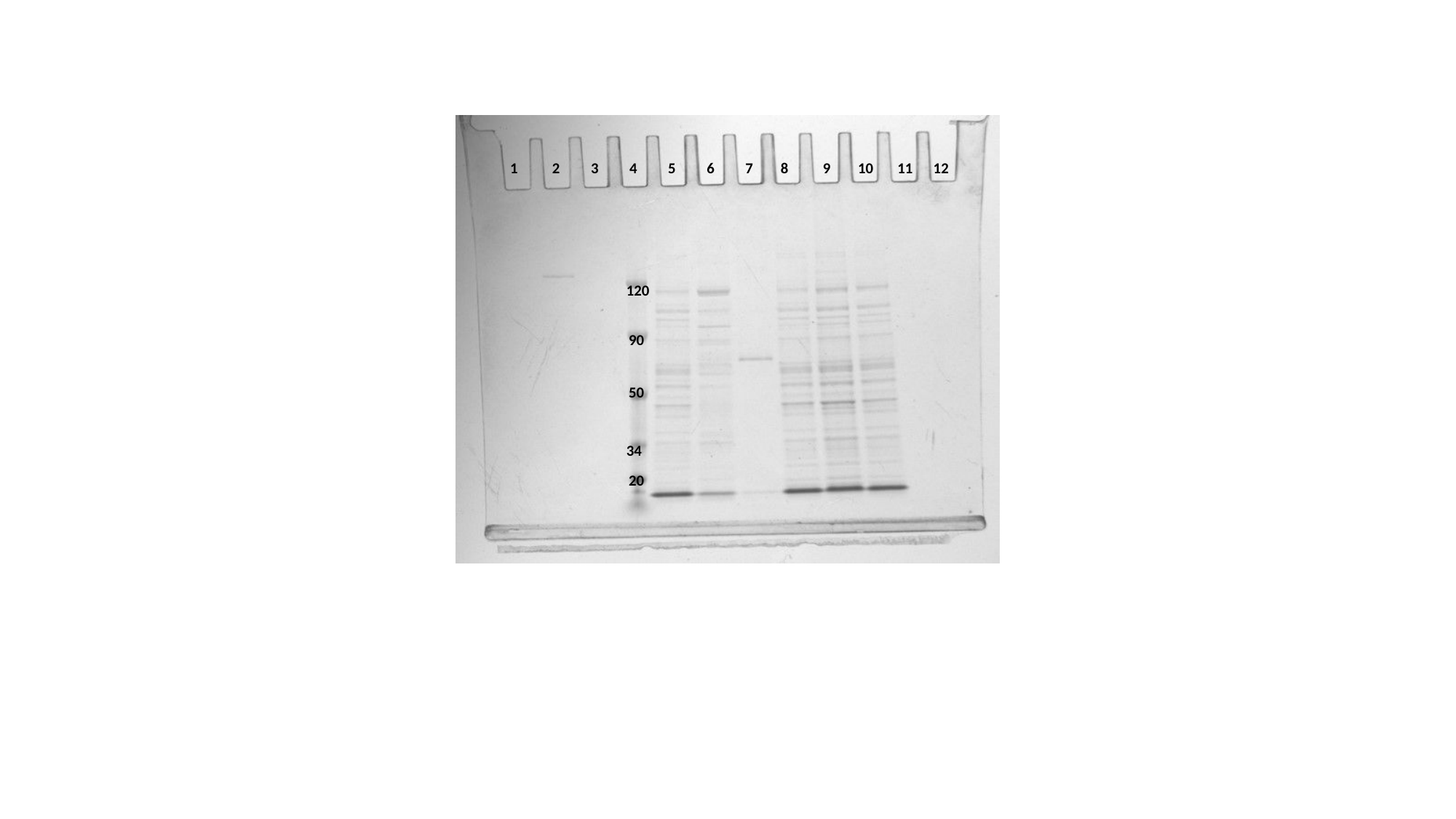

120
90
50
34
20
 1 2 3 4 5 6 7 8 9 10 11 12

Supplement: Supplementary file 1 — Additional file 1: Figure S1. Representative SDS-PAGE gel electrophoresis of samples from purification of recombinant N. meningitidis serogroup W capsule polymerase. Lanes 2 and 3 are samples from the eluate. Lane 4 is the molecular weight marker containing proteins of the specified molecular weights. Lanes 5–7 are samples from the column wash steps. Lane 8 contains column flow through, Lane 9 is the cell lysate and Lane 10 is the supernatant. Lanes 1, 11, and 12 were not loaded with sample. [file 13104_2018_3596_MOESM1_ESM.pptx]

## Slide 1
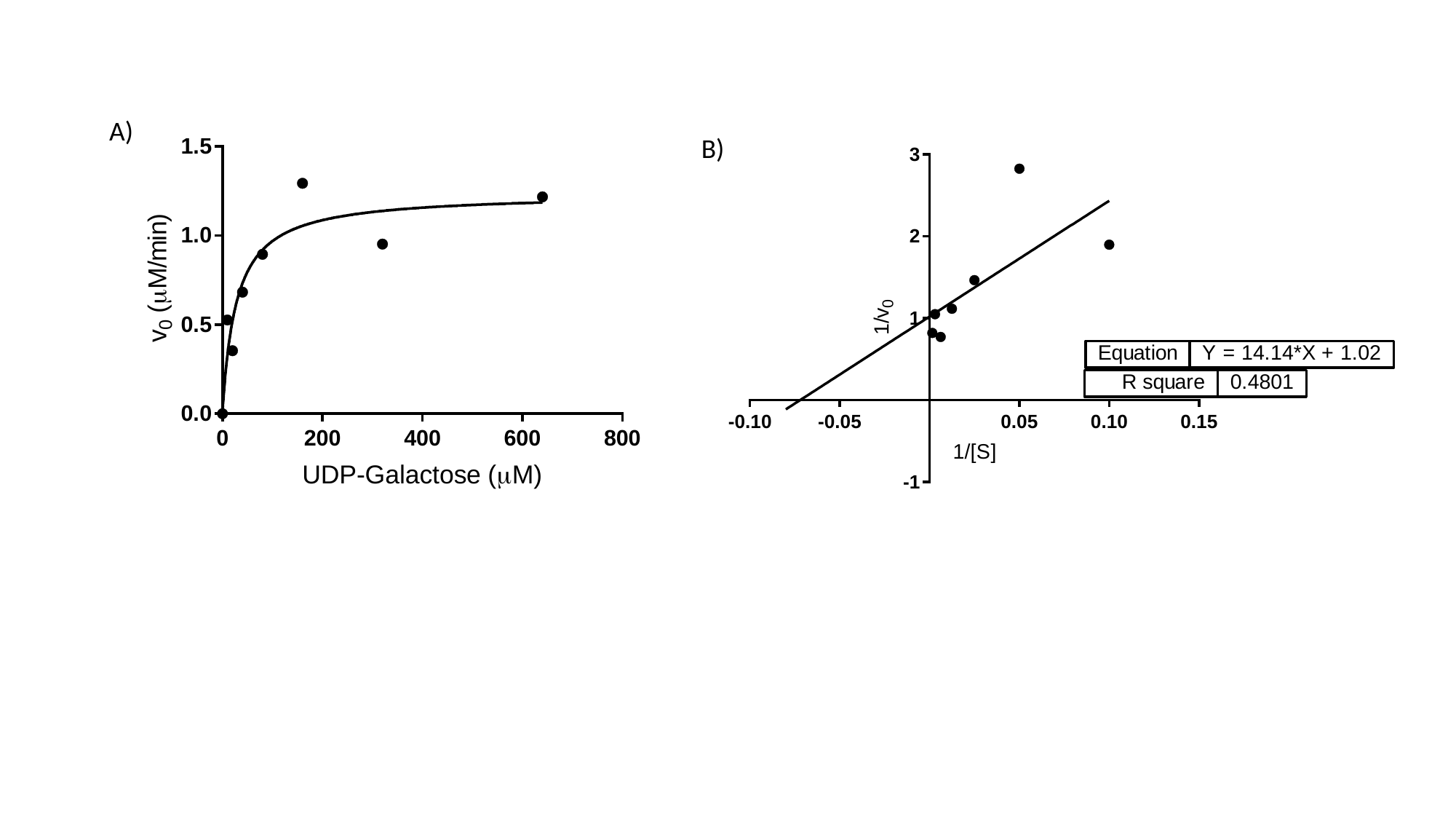

A)
B)

Supplement: Supplementary file 3 — Additional file 3: Figure S2. Kinetic effects of varying UDP-galactose concentrations (10–640 µM) on UDP-galactosyltransferase activity of the N. meningitidis serogroup W capsule polymerase. Reactions were performed in duplicate and the averages are plotted. A) Michaelis-Menten plot and B) Lineweaver-Burke plot. [file 13104_2018_3596_MOESM3_ESM.pptx]

## Slide 1
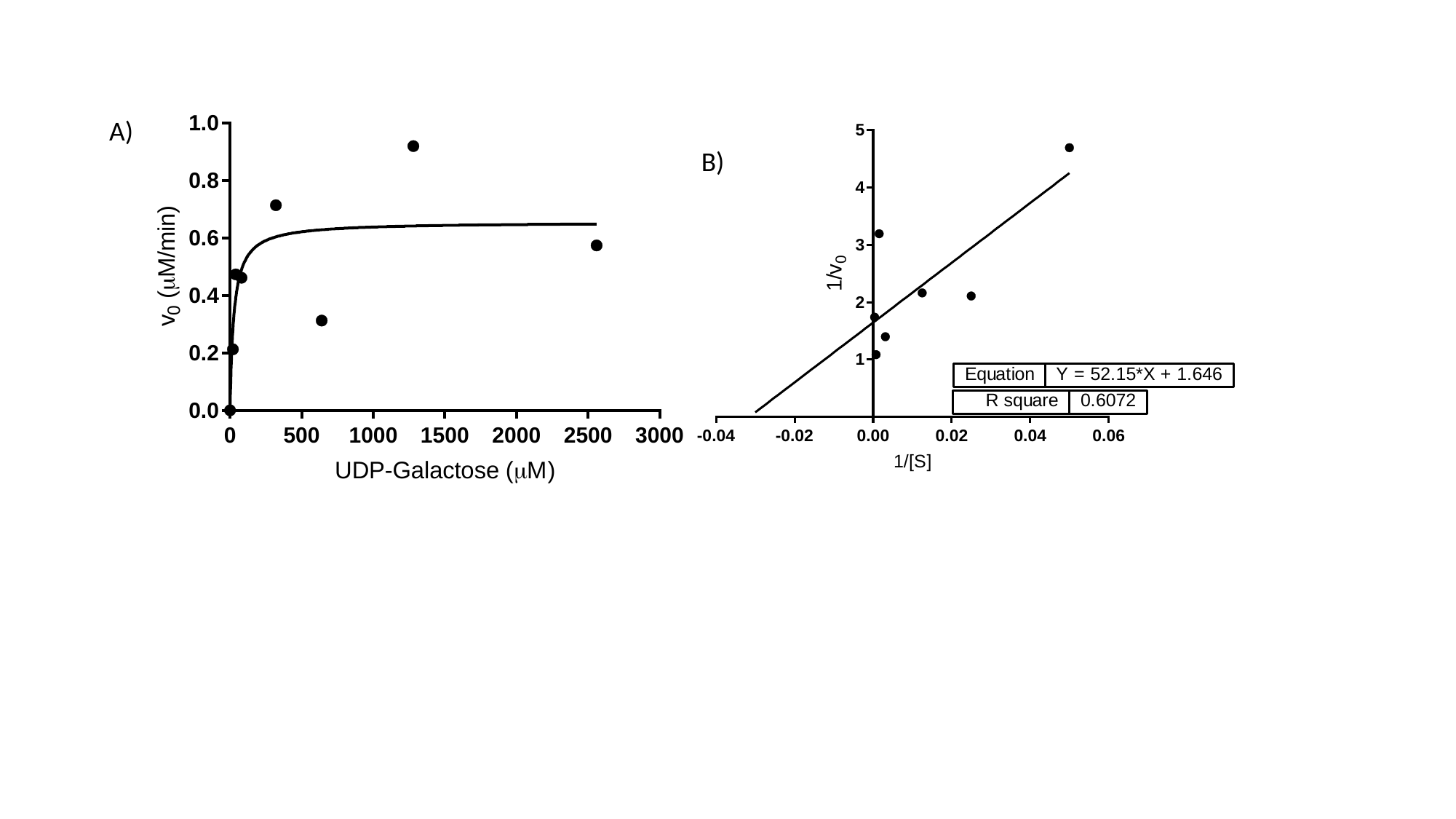

A)
B)

Supplement: Supplementary file 4 — Additional file 4: Figure S3. Kinetic effects of varying UDP-galactose concentrations (20–2560 µM) on UDP– galactosyltransferase activity. This data includes the potential outlier (640 µM). Reactions were performed in duplicate and the averages are plotted. A) Michaelis-Menten plot and B) Lineweaver-Burke plot. [file 13104_2018_3596_MOESM4_ESM.pptx]
