# Supplementary material for: Determination of the binding affinities of Neisseria meningitidis serogroup W capsule polymerase with two nucleotide sugar substrates
Source: BMC Res Notes. 2018 Jul 16;11:482. doi: 10.1186/s13104-018-3596-y (PMC6048754; doi:10.1186/s13104-018-3596-y)
Supplement: Supplementary file 2 — Additional file 2: Methods. Absorbance-based multi-enzyme coupled activity assay. Describes the conditions of the assay for data produced in Additional file 3: Figure S2 and Additional file 4: Figure S3 [file 13104_2018_3596_MOESM2_ESM.docx]

**Additional file 2: Methods**

*Absorbance-based multi-enzyme coupled activity assay*

In one set of reaction conditions, polysaccharide (56 µg/mL), 0.7 mM PEP, 2 mM ATP, 7.2 units PK/LDH, 0.15 mM NADH, 2 mM CMP-sialic Acid, UDP-galactose (at 10, 20, 40, 80, 160, 320 or 640 µM), 1 mM DTT, and 50 mM Tris, 25 mM MgCl_2_ (pH 8.0) in the absence (control reactions) or presence of serogroup W capsule polymerase (10 µg/mL). The average rates of each reaction were calculated and plotted using GraphPad Prism. Similarly, another set of reactions were performed with the same conditions, 2 mM UDP-galactose and .05 units/mL NMPK with 10, 20, 40, 80, 160, 320 or 640 µM CMP-sialic acid (Nacalai Tesque). All reactions were performed at room temperature in duplicate in a final volume of 800 µL. Similar reactions were also performed in the same manner keeping CMP-Sialic Acid constant at 2 mM and varying UDP-galactose. Samples were equilibrated for 5 min before initiation of the reaction. Absorbance was monitored at 340 nm for 20 min using a Cary 50 spectrophotometer. The rates of reaction were calculated using the following equation (((Slope – Background)/ 2) / 6220 M^-1^ cm^-1^) x 10^6^.

In another set of reactions, similar conditions were used with the described modifications. The nucleotide donor sugar concentrations used were 20, 40, 80, 320, 640,1280, and 2560 μM. Acceptor was increased to a concentration of 625 µg/mL and the serogroup W enzyme was 25 µg/mL. The final volume of these reactions was 200 µL and absorbance was monitored for 10 min.
